# Smartphone-based digital phenotyping for characterizing post-operative recovery in patients undergoing surgery for cervical myelopathy

**DOI:** 10.3389/fneur.2026.1694719

**Published:** 2026-03-19

**Authors:** Denise Feurer, Luca Vedovelli, Fabio Moscolo, Christian Soda, Francesco Sala, Dario Gregori, Alessandro Boaro

**Affiliations:** 1 Unit of Biostatistics, Epidemiology and Public Health, Department of Cardiac, Thoracic, Vascular Sciences and Public Health, University of Padova, Padova, Italy; 2 Neurosurgery Unit, Carlo Poma Hospital, Mantova, Italy; 3 Neurosurgery Unit, Azienda Ospedaliera Universitaria Integrata, Verona, Italy; 4 Section of Neurosurgery, Department of Neurosciences, Biomedicine and Movement Sciences, University of Verona, Verona, Italy

**Keywords:** cervical myelopathy, digital phenotyping, neurosurgery, smartphone, spinal cord

## Abstract

**Objective:**

This study aimed at investigating the feasibility of using personal smartphones to characterize mobility in patients after surgery for cervical myelopathy. The specific objectives are to (1) assess differences in global positioning system (GPS)/accelerometer mobility features between the first and fifth post-operative weeks, (2) evaluate differences in recovery trajectories between anterior and posterior surgical approaches, and (3) analyze correlations between pain/disability scores and passively acquired GPS/accelerometer mobility variables.

**Methods:**

A population of patients with cervical myelopathy undergoing surgical decompression at the Verona University Hospital was enrolled in this study. Data collection included passively acquired GPS and accelerometer data from personal smartphones, patient-reported outcome measures (PROMs), and demographic/surgical information. Statistical analysis included descriptive statistics, Wilcoxon rank sum tests for comparing mobility variables between the first and fifth post-operative weeks, generalized linear models to assess recovery trajectories, and the Pearson’s correlation to evaluate relationships between mobility variables and PROMs.

**Results:**

A total of 13 patients (3 female and 10 male patients) were included. All five mobility variables showed significant improvement from post-operative week 1 to week 5 (*p* < 0.001). The surgical approach was significantly associated with recovery trajectories for *Home Duration*, *Steps*, and *Significant Location Count*. Patients who underwent anterior surgery generally showed higher initial mobility levels and steeper recovery trajectories. The visual analog scale (VAS), the modified Japanese Orthopedic Association (mJOA), and the Oswestry Disability Index (ODI) were significantly correlated with all five GPS variables, whereas the Neck Disability Index (NDI) was only significantly correlated with *Distance Traveled* and *Steps*. Linear relationships between mobility variables and PROMs were observed, with increasing uncertainty at higher mobility levels.

**Conclusion:**

The study demonstrates the potential of smartphone-derived mobility data as a valuable tool for characterizing post-operative recovery in cervical myelopathy patients. The ability to characterize recovery trajectories and correlations with established clinical measures indicates that digital phenotyping could complement traditional assessment methods, offering continuous, objective data to support clinical decision-making and personalized patient care.

## 1 Introduction

Cervical myelopathy is defined as chronic damage to the spinal cord caused by progressive compression forces exerted by degenerative changes in the cervical spine elements or by slowly growing oncological diseases (1). It is a significant cause of disability worldwide, with symptoms that include gait disturbances, neck pain, arm and hand weakness, and numbness (2, 3). Successful surgical interventions can prevent further clinical progression and optimize the quality of life (3). The indication for surgery relies on an accurate medical history and physical examination, along with appropriate imaging.

The success of surgical treatment for cervical myelopathy is typically evaluated during follow-up visits, when patients complete questionnaires about their pain levels, self-care abilities, and capacity to perform physical activities and work. However, these approaches are limited in their ability to provide continuous and objective monitoring of patient recovery (4).

There is a need for more objective and patient-centered tools to assess recovery after surgery. Recent research has focused on the use of smartphones and wearable devices to collect this information. This approach, known as digital phenotyping, has been defined as the moment-by-moment quantification of the individual-level human phenotype *in-situ* using data from smartphones and other personal digital devices (5–7). Digital phenotyping utilizes data streams primarily from personally owned digital devices, eliminating the need for additional wearable devices for data collection. Smartphone-derived data can be categorized into two types: active and passive. Active data include user-initiated inputs such as surveys and voice recordings, while passive data encompass sensor information (e.g., accelerometer and gyroscope) and usage metrics (e.g., communication logs and screen activity) (8, 9).

This methodology has recently demonstrated success in characterizing behavior and functional status across various neurosurgical patient populations, highlighting its potential for developing clinically valid follow-up tools (10–13). Integrating passive data with established patient-reported outcomes measures (PROMs) could potentially validate smartphones as non-invasive, scalable instruments for patient monitoring and quality-of-life assessment.

This study aimed to investigate the feasibility of using personal smartphones to accurately characterize the mobility of patients after surgery for cervical myelopathy. We hypothesized that daily metrics derived from smartphone global positioning system (GPS) and accelerometer data could effectively characterize patients’ post-operative physical recovery trajectory. We focused on three objectives to test the usability of this system to measure the progress during the recovery period: (1) to assess the differences in GPS/accelerometer mobility features between the first and the fifth post-operative weeks, (2) to assess the differences in recovery trajectory between anterior and posterior surgical approaches, and (3) to evaluate the correlation between different pain/disability scores and passively acquired GPS/accelerometer mobility variables.

In the future, this approach may be used to evaluate recovery trajectories for cervical myelopathy patients, providing valuable information for patient counseling and rehabilitation decision-making. This data-driven approach could lead to more personalized and effective treatment strategies for individuals with cervical myelopathy.

## 2 Materials and methods

### 2.1 Patient population

This study was approved by the Ethics Committee and reviewed under the protocol number 3673CESC. Patient enrolment was conducted at the Department of Neurosurgery of the Verona University Hospital. Eligible patients with radiological and clinical evidence of cervical myelopathy and an indication for surgical decompression were invited to participate in the study. Patients who provided consent were asked to download and activate the Beiwe smartphone application, which serves as the front-end of the research platform used for data collection and analysis (14). The Beiwe application, available for both Android and iOS devices, collects both active and passive data and is accessible only through a unique username and password known exclusively to the user. The recent version of the application captures multiple data streams, including GPS data, accelerometer measurements, Bluetooth and Wi-Fi information, phone and call logs and screen on/off activity. These data streams are captured at customizable sampling frequencies. For this study, Beiwe was configured to collect GPS data for 1 min every 5 min and accelerometer data for 10 s every 10 s. All smartphone data collected using Beiwe were immediately encrypted on the device. The data were then uploaded to the Beiwe back-end system based on a university-owned Amazon Web Services (AWS) cloud, in compliance with the General Data Protection Regulation. Only the server, which has a private key, can decrypt the collected data, ensuring that the data stored by the app cannot be compromised. For additional information please refer to the documentation at https://github.com/onnela-lab/beiwe-backend. Patients were excluded *a priori* if they did not possess an iOS or Android phone or if they were under 18 years of age. By protocol, data were acquired and patients were prospectively followed for 3 months.

### 2.2 Data collection

Three types of data were collected for the purposes of this study: passively acquired GPS and accelerometer data, patient reported outcome measures (PROMs) on pain and physical disability (15) and basic demographic and surgical approach information.

GPS and accelerometer data were produced as daily summary statistics and included five different features: *Distance Traveled*, *Significant* *Location Count*, *Significant Location Entropy*, *Home Duration*, and *Steps* (Table 1). PROMs included visual analog scale (VAS) for pain (16), modified Japanese Orthopedic Association (mJOA) score (17), Oswestry disability index (ODI) (18, 19), and neck disability index (NDI) (20) (Table 1). The VAS for pain was collected on a daily basis, while the other three scores were collected on a weekly basis in order to avoid questionnaire fatigue. The inclusion of these four outcome measures allows a complete characterization of the patient condition, taking into consideration pain related to intrinsic pathological condition as well as the surgical intervention, symptoms specifically due to myelopathy, and the level of disability in conducting daily activities. Basic demographics and surgical approach variables included age, gender, cervical spine level of disease, anterior verses posterior surgical approach, type of surgery, and presence of instrumentation.

**Table 1:** GPS, accelerometer, and patient reported outcomes variables.

Variable	Description of variable	Description of measurement
GPS and accelerometer data		
*Distance traveled*	Total distance traveled over the course of a day (in km)	The sum of lengths of all flights. A flight is defined to be the longest straight-line trip of a particle from one location to another without a directional change or pause
*Significant location count*	Number of significant locations visited at any point of a day	Significant locations are distinct pauses which are at least 15 min long and 50 meters apart. They are determined using K-means clustering on locations that a patient visits over the course of a follow-up.
*Significant location entropy*	Entropy measure based on the proportion of time spent at significant locations over the course of a day	Letting p_1 be the proportion of the day spent at significant location I, significant location entropy is calculated as as -\sum_{i} p_i\log(p_i), where the sum occurs over all non-zero p_i for that day.
*Home Duration*	Time spent at home over the course of the day (in hours)	“Home” is the most frequently visited significant location for a person between the hours of 8 pm and 8 a.m. each day over the course of follow up
*Steps*	Number of steps taken	Obtained from the smartphone accelerometer data
Patient reported outcome measures		
VAS (Visual Analogue Scale) scale for pain	Measures pain intensity	10-point scale. Min: 0 (no pain) – Max: 10 (worst pain imaginable)
mJOA (modified Japanese orthopaedic association) score	Measures baseline myelopathy severity, postoperative improvements and social independence	18-point scale Min: 0–11(severe myelopathy) – Max: 15–17 (mild myelopathy)
ODI (Oswestry disability index)	Assesses a patient’s level of disability in activities of daily living	50-point scale converted to percentages. Min: 0–20% (minimal disability) – Max: 81–100% (complete disability)
NDI (Neck disability index)	Assesses self-rated disability in patients with neck main.	50-point scale converted to percentages Min: 0–8% (no disability) – Max: 70–10% (complete disability)

### 2.3 Data cleaning and preparation

Data preprocessing was performed through a series of steps. First, patterns of missingness were assessed across all mobility-related variables. Observations beyond the 70-day post-operative window were censored to standardize follow-up duration, and any variable with more than 50% missing values within a patient’s records was excluded from further analysis. To address remaining missing values, we applied multiple imputation by chained equations (MICE) (21) using a random-forest–based imputation model to generate five complete datasets over 50 iterations, with a fixed seed to ensure reproducibility. The imputed datasets were then consolidated into a single analytic dataset. Univariate outliers were detected through graphical inspection and subsequently removed. Variables exhibiting marked deviation from normality were log-transformed to stabilize variance and approximate a Gaussian distribution. Patient-reported outcome measures (PROMs) were analyzed using all available non-missing observations. All data-cleaning and imputation procedures were executed in R through the mice package, with the *complete()* function used to pool the imputed datasets.

### 2.4 Statistical analysis

Using the prepared database, we conducted a descriptive analysis of the patients to provide an overview of their age, sex, and surgical approach. To address the first study objective, we calculated the mean values of the log-transformed mobility variables for post-operative weeks 1 and 5 and compared them using the Wilcoxon rank sum test. We used a generalized linear model (GLM) approach as a unique framework to model the outcomes of interest. We implemented a homoscedastic Gaussian GLM to assess the linear recovery trajectory across different mobility. For *Distance Traveled*, *Home Duration,* and *Significant Location Entropy,* we used a Gamma distribution, and for *Significant Location Count* and *Steps,* we used a Poisson distribution (log link). In the GLM, we added an interaction term in the linear predictor (Postop Day * Surgery Approach) to identify different outcomes, as the surgery approach may change the post-operative recovery. Finally, to address the third objective of the study, we used the Pearson’s correlation to measure the linear relationship between the mobility variables and PROMs. We additionally modeled the relationship between the variables using a GLM for visual presentation. (Supplementary File 1 includes additional information on GLM distributions, effect sizes, and confidence intervals).

## 3 Results

### 3.1 Descriptive results

In total, 13 patients were included, consisting of three female and ten male patients (Table 2). The majority of patients underwent surgery on one or two cervical levels (77%). The surgical approach largely depended on patient age (Table 3). Six patients with a mean age of 50 years had surgery through an anterior approach, while seven patients, with a mean age of 67 years underwent surgery through a posterior approach. Additional details regarding patient population and types of surgery are presented in Table 2. With regards to the mobility-based dataset, a total of 4,545 observations for each of the five mobility variables were included after data cleaning and multiple imputation. For PROMs, we collected 460 daily surveys for VAS, 92 weekly surveys for mJOA, 90 for NDI, and 96 for ODI.

**Table 2:** Overview of patients’ characteristics.

Patient characteristics		
Gender	M	10
	F	3
Mean age (years)		67
	
Spine levels involved	1	4
	2	6
	3	2
	4	1
Surgical approach	Anterior	6
	Posterior	7
Instrumentation	Yes	9
	No	4

**Table 3:** Correlation between surgical approach with age and gender.

Variable	Value	Surgery approach				*p*-value
		Anterior (*n* = 6)		Posterior (*n* = 7)		
		Mean	95% CI	Mean	95% CI	
Age		50	42.5, 57.5	66.8	59.8, 73.9	0.01
		*N*	Percentage	*N*	Percentage	*p*-value
Gender	Female	2	33.30%	1	14.30%	0.88
	Male	4	66.70%	6	85.70%	

### 3.2 Objective 1: post-operative week comparison

All five mobility variables showed significant improvement from post-operative week 1 to post-operative week 5, with *p*-values of <0.001 (Table 4). *Distance Traveled* increased from 3 km (95% CI: 2.3–3.7) to 14 km (95% CI: 12–16), and *Steps* increased from a count of 109 (95% CI: 91–127) to 250 (95% CI: 223–277). The *Significant Locations* variables increased in both *Count* and *Entropy*, from 1.33 counts (95% CI: 1.3–1.4) to 2.44 counts (95% CI: 2.3–2.6) and from 0.10 entropy (95% CI: 0.08–0.12) to 0.26 entropy (95% CI: 0.23–0.29), respectively. *Home Duration* decreased slightly, from 15 h (95% CI: 14–16) to 14 h (95% CI: 13–15).

**Table 4:** Comparison of GPS and accelerometer variables at postoperative week 1 and 5.

Variable	Postop week				*p*-value
	Week 1 (*N* = 455)		Week 5 (*N* = 450)		
	Mean	95% CI	Mean	95% CI	
*Distance traveled*	3.04	2.34, 3.73	13.89	12.17, 15.60	< 0.001
*Home duration*	14.63	13.62, 15.64	14.05	13.13, 14.98	< 0.001
*Significant location count*	1.33	1.26, 1.40	2.44	2.28, 2.61	< 0.001
*Significant location entropy*	0.1	0.08, 0.12	0.26	0.23, 0.29	< 0.001
*Steps*	108.83	90.70, 126.96	250	222.88, 277.13	< 0.001

### 3.3 Objective 2: linear recovery of anterior and posterior surgery patients

**Figure 1:**
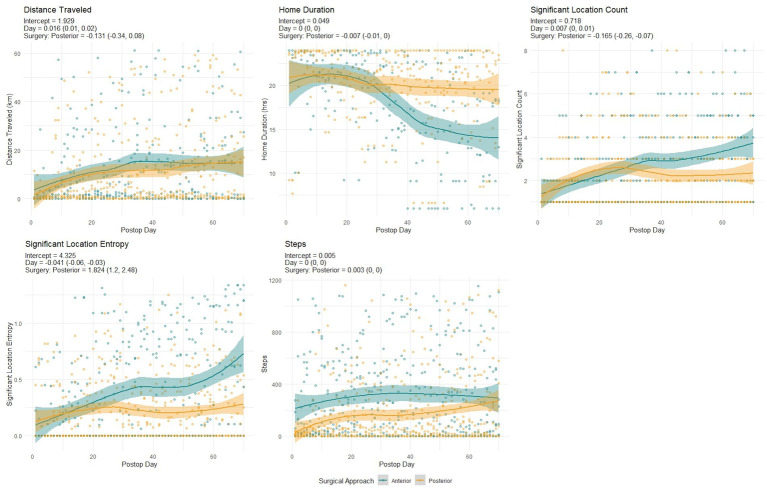
Recovery trajectories for the anterior and posterior surgery approaches across different recovery variables (distance traveled, home duration, significant location count, significant location entropy, steps). The data points, the fitted curve, and the confidence band for the anterior surgery group are in green, the ones for the posterior surgery group are in yellow.

The surgery approach was significantly associated with the recovery trajectory in terms of *Home Duration*, *Steps,* and *Significant Location Count*. *Distance Traveled* was not significantly associated, and the trajectories for this variable were similar for both surgery approaches (Figure 1). While *Significant Location Entropy* was also not significantly impacted by the surgical approach, the values and trajectories for the anterior group were generally higher and steeper than the posterior group.

*Home Duration* decreased slightly for the anterior surgical group but increased for the posterior group. *Significant Location Count* had a moderately strong association with the surgical approach, with a slightly steeper recovery for the anterior group. The association for *Steps* was strongly significant; the anterior group had a higher count initially but a relatively flat recovery trajectory, while the posterior group started much lower but had a steeper increase in counts, reaching almost similar levels to the anterior group after 10 weeks of recovery.

### 3.4 Objective 3: correlation between mobility variables and pain/disability scores

**Figure 2:**
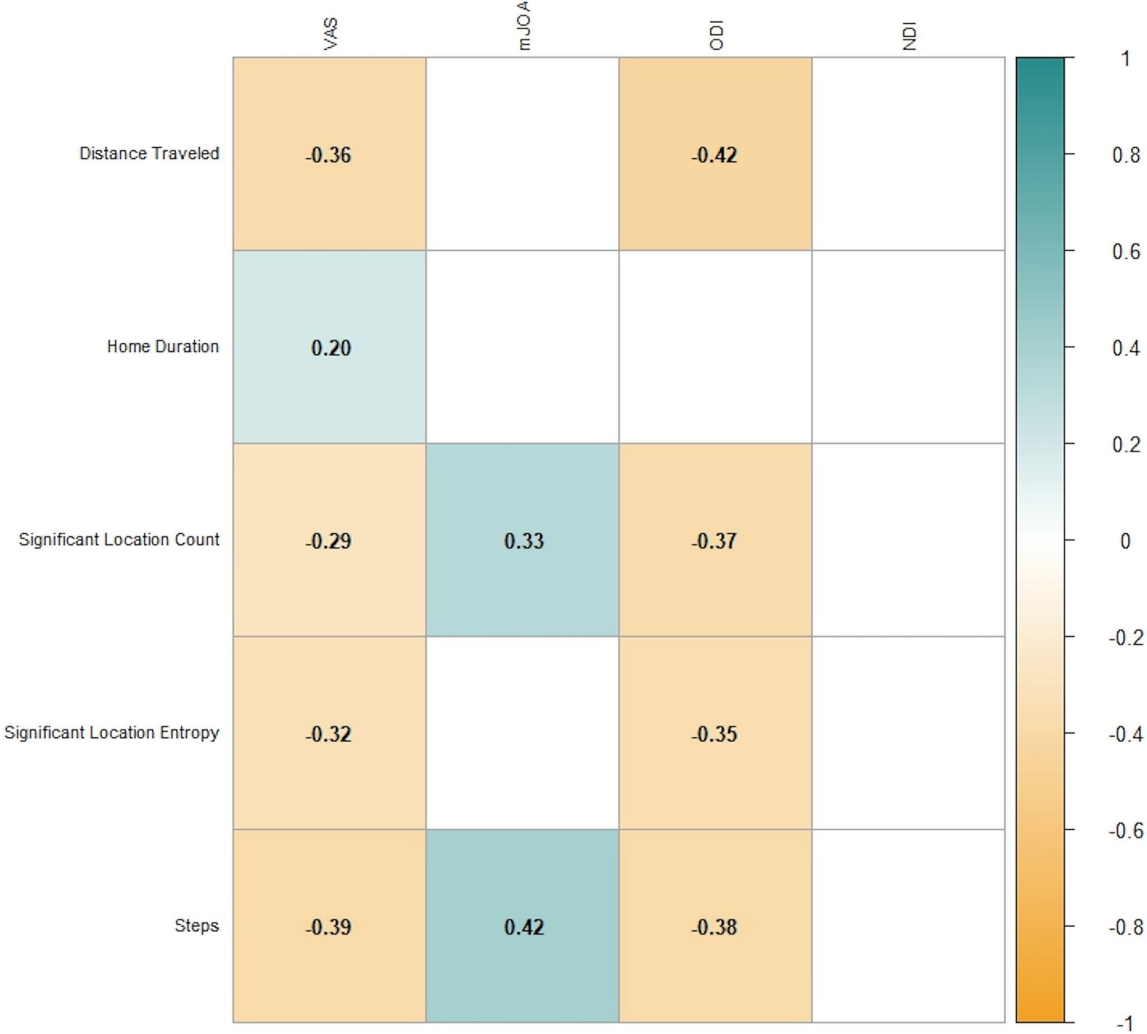
Statistically significant correlations (*p* < 0.5) between mobility variables and pain/disability variable. VAS: Visual analogic scale for pain; mJOA: modified Japanese orthopedic association score; ODI: Oswestry disability index; NDI: neck disability index.

VAS, mJOA, and ODI were all significantly correlated with each of the five GPS variables, while NDI was only significantly correlated with *Distance Traveled* and *Steps* (Figure 2).

VAS had a moderately strong correlation with *Distance Traveled* (−0.358) and *Steps* (−0.369). MJOA had a moderately strong correlation with *Steps* (0.403), *Significant Location Entropy* (0.407), and *Significant Location Count* (0.359). ODI had a moderately strong correlation with *Distance Traveled* (−0.409), *Significant Location Entropy* (−0.361), *Significant Location Count* (−0.351), and *Steps* (−0.358). NDI did not exhibit any strong correlations, with the highest correlation observed for *Distance Traveled* (−0.279). *Home Duration* was the mobility variable that was least correlated with any of the PROMs, with the highest correlation observed for VAS (0.227). We conducted multiple comparisons correction using false discovery rate (FDR), which confirmed the significance in all cases with the exception of the *Significant Location Entropy* – mJOA correlation (*p*-values from 0.037 to 0.061).

**Figure 3:**
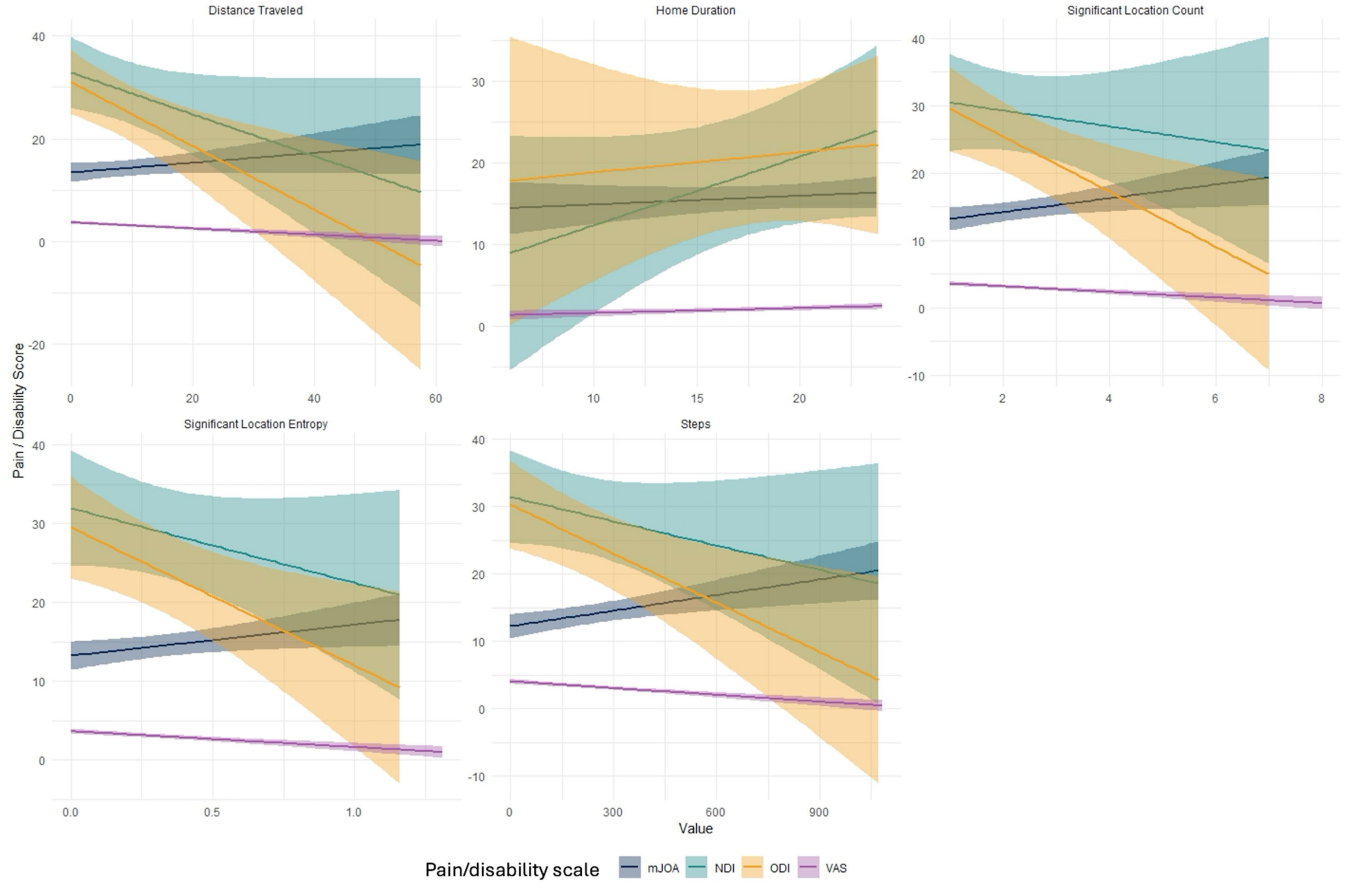
Linear relationships between mobility variables and pain/disability scores. Fitted curve and confidence bands are in blue for mJOA, green for NDI, yellow for ODI, and pink for VAS.

Figure 3 shows the linear relationship between each mobility variable and the PROMs. MJOA scores increased consistently with increasing values of the mobility variables, except for *Home Duration*, which demonstrated an inverse relationship. The other PROM scores exhibited an inverse correlation with the same mobility variables; however, NDI and ODI demonstrated greater uncertainty. Overall, the uncertainty for all the PROM scores increased with increasing values of the mobility variables.

## 4 Discussion

The indications and optimal timing for surgical intervention in degenerative cervical myelopathy (DCM) remain uncertain, underscoring the need for additional high-quality real-world data encompassing the full spectrum of disease severity. The current clinical guidelines recommend surgical treatment for patients with moderate to severe or progressive DCM in order to prevent further neurological deterioration. Prospective, multicenter, and registry-based studies have demonstrated that, in appropriately selected patients, decompressive surgery can arrest disease progression and lead to clinically meaningful—though often modest—improvements in pain, functional status, and quality of life (22–24). The characterization of such post-operative improvements can be primarily assessed through the use of PROMs that have limitations in their ability to provide an objective characterization of patient recovery, and there is no standardized use for them across clinicians.

In this study, we investigated the use of smartphone-derived mobility data to characterize recovery trajectories in patients undergoing surgery for cervical myelopathy. The findings provide valuable insights into the potential of digital phenotyping to assess post-operative recovery and its correlation with traditional pain and disability measures. In addition, the findings of previous studies on the characterization of post-operative mobility in different spinal conditions contribute to the validation of personal smartphones as robust and efficient monitoring tools (10, 11).

The significant improvement observed in all five mobility variables from post-operative week 1 to week 5 indicates that smartphone-derived data have the potential to capture recovery trajectories. Compared with previous studies, the *Distance Traveled* in the post-operative period showed overall reduced values, which may be attributable to the origin of the patient population. In Boaro et al. (10) the patient cohort was based in the United States, where the distances covered for health-related reasons are, on average, greater than in Italy; the patient population of this study originated from the Veneto region alone (area of 18.300 km^2^). Regardless of these differences, in both studies, the *Distance Traveled* covered daily increased with time after surgery, supporting the robustness of the approach. *Time spent at (Home Duration)*, *significant location entropy* and *count* showed similar values and trends across studies and spine conditions (10, 11). We were more cautious in assigning a strong significance to the *Step Count* values, despite the observed positive trend, as an accurate estimate of *Steps* requires the assessment of spatial gait features at a single patient level, which was not performed in this study.

The differential recovery trajectories observed between the anterior and posterior surgical approaches are particularly interesting. Patients who underwent anterior surgery generally showed higher initial mobility levels and steeper recovery trajectories, especially in terms of *Significant Location Count* and *Steps*. Such findings could be attributed to the less invasive nature of the anterior approach, potentially allowing for quicker initial recovery; however, the anterior approach population was significantly younger than the posterior approach population; therefore, age could act as a confounding factor explaining a faster recovery in younger patients. Similarly, *Home Duration* presented an increase rather than a decrease in the values in the older, posterior approach population; such findings could be at least partially explained by age as well. However, the convergence of mobility levels between the two groups over the course of the weeks suggests that the long-term functional outcomes are similar, regardless of the surgical approach (25, 26).

The correlations between mobility variables and traditional pain/disability scores (VAS, mJOA, ODI, and NDI) provide additional evidence for the concurrent validity of smartphone-derived measures. Cote et al. (11) first reported an inverse relationship between VAS for pain and smartphone-derived mobility measures in a patient population with spine disease. Although this relationship may seem intuitive, it has been traditionally based on patients’ qualitative reports of their own clinical condition, which are subject to personal filters and recall bias. Such observation opened the way to expand the use of digital phenotyping for a more objective characterization of mobility. Boaro et al. (10) explored the relationship between additional PROMs characterizing different aspects of patients’ functional status and passively acquired mobility measures, observing stronger associations between questionnaires characterizing physical aspects of quality of life than with psychological and social aspects. In both these articles, the study population included different types of spine pathologies, while, in our study, we primarily focused on a single pathological entity in order to provide a more tailored characterization of this relationship. Moderately strong correlations were observed, particularly with VAS, mJOA, and ODI, suggesting that digital phenotyping measures may have the potential to serve as objective proxies for patient-reported outcomes in patients undergoing surgery for cervical myelopathy. Most notably, the correlations observed in this study tended to be smaller than those reported by Boaro et al., a difference that potentially resides in the difference in pain level and disability status between lumbar and cervical pathologies (10). Cervical myelopathy, particularly in its mild form, does not present pain as a main symptom, and physical symptoms take a long time to develop. In contrast, lumbar herniated disc—the most frequent diagnosis in Boaro et al.—showed a high level of acute pain and perceived and actual disability, which are both readily addressed and often resolved by a surgical intervention, translating in stronger correlations with post-operative mobility measures. NDI showed weaker correlations with mobility variables compared to other measures, which is unsurprising, as neck pain and disability related to neck symptoms are not typically the main complaints reported by cervical myelopathy patients. Neck pain may be present after surgery, specifically in patients who underwent a posterior approach, where the strong posterior neck muscles and tendons are cut and dissected to access the cervical spine, and this may be reflected both in the VAS scores and in the weaker, although present, correlation of the NDI scores with mobility measures.

The linear relationships between mobility variables and pain/disability scores further support the potential of digital phenotyping in monitoring recovery. The consistent positive relationship between mJOA scores and mobility measures aligns with the expected improvement in neurological function following surgery. The inverse relationships observed with VAS, NDI, and ODI also logically correspond to decreasing pain and disability as mobility improves.

However, the increasing uncertainty in these relationships at higher mobility levels suggests that, beyond a certain point, improvements in mobility may not necessarily translate to proportional improvements in pain or disability scores (27). This finding underscores the complex nature of recovery and the need for a multifaceted approach to patient assessment. The literature on recovery after surgery for cervical myelopathy primarily focuses on an extended follow-up period of up to 12 months, which is generally considered the time needed to obtain stable, long-term improvements. In our population, over the course of 5 weeks, the most evident changes were observed in VAS, with a reduction from 5 to 2.8 points, and in ODI, with a reduction from 40 to 17%; the improvements were less evident in the mJOA, which presented an average improvement of 0.5 points, largely driven by the anterior surgery group (1.3 points on average), and in the NDI, which presented an initial value of 28.4, an erratic trend in the weeks from 2 to 4 and a final value of 16.9. The values of ODI and NDI improved beyond the minimal clinically important differences (MCIDs) of 5–10 points generally observed in the literature for spine patients, while only the anterior surgery group presented a clinically relevant difference in mJOA (MCIDs for mJOA is between 1 to 2 points) (28–30). Our observations on the change in PROMs scores and mobility measures over the course of the first 5 post-operative weeks are difficult to compare with more traditional studies, but they suggest the readiness of the spinal cord to initiate an albeit partial recovery process. The readiness of the nervous structures to recover after resolution of chronic compression is also supported by neurophysiological studies, which observed the most improvements in neurophysiological parameters assessed by transcranial magnetic stimulation occur within the first 6 months after surgery (31).

Several important limitations should be considered when interpreting these findings. First, patients who did not own a smartphone were excluded *a priori*, potentially introducing a selection bias toward specific groups; second, the small sample size limits the generalizability of the results, given that the clinical presentation of cervical myelopathy can be highly variable, particularly in terms of symptoms intensity. Related to this point, the limitation in the assessment of feasibility of the measurements themselves is partially mitigated by the fact that the same smartphone technology has been used in previous studies (8–11), supporting the robustness of a digital phenotyping approach across different pathologies. In addition, although our study population was older compared to previous similar studies (10), which might impact the ability to use a personal smartphone, we observed neither dropouts nor cases of complete data absence. Third, as highlighted above, the significance of the *Steps* is limited, since accurate estimation requires patient-level spatial gait analysis, which was not performed in this study. Finally, we measured data originating from personal smartphones, which act as proxies for actual patient activity. Alongside these limitations, several strengths should also be highlighted. First, the usage of smartphone-derived data enables effortless collection of multiple data types simultaneously. Second, smartphone applications are resourceful, as most patients already own smartphones, eliminating the need for additional, expensive monitoring equipment.

In conclusion, this study demonstrates the potential of smartphone-derived mobility data as a valuable tool for monitoring post-operative recovery in cervical spine surgery patients. The ability to characterize recovery trajectories and their correlations with established clinical measures suggests that digital phenotyping could complement traditional assessment methods, offering continuous, objective data to support clinical decision-making and personalized patient care. Future research should focus on larger, more diverse patient populations and longer follow-up periods to further validate these findings and explore the long-term utility of digital phenotyping in spine surgery outcomes.

## Data Availability

The raw data supporting the conclusions of this article will be made available by the authors upon request, without undue reservation.
