# Supplementary material for: Smartphone-based digital phenotyping for characterizing post-operative recovery in patients undergoing surgery for cervical myelopathy
Source: Front Neurol. 2026 Mar 19;17:1694719. doi: 10.3389/fneur.2026.1694719 (PMC13043427; doi:10.3389/fneur.2026.1694719)
Supplement: Supplementary file 1 [file Supplementary_file_1.docx]

Supplementary File 1

Diagnostic plots for the GLM distributions are reported below.

Distance Travelled


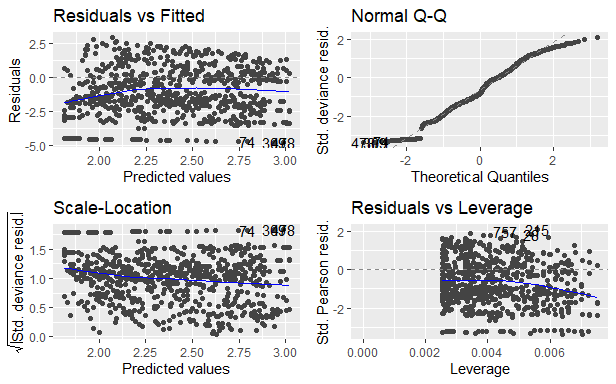


Home duration


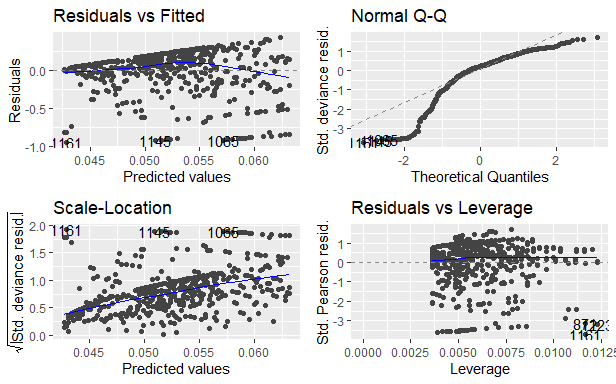


Significant location count


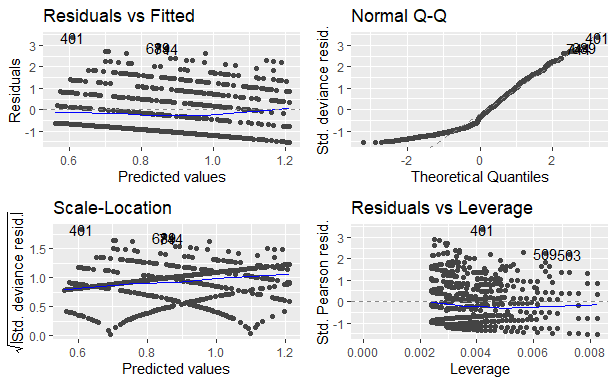


Significant location entropy


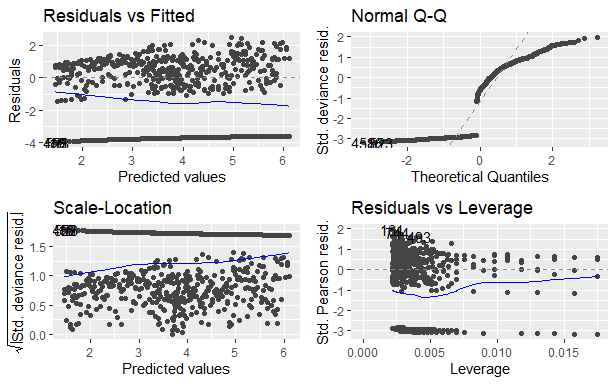


Steps


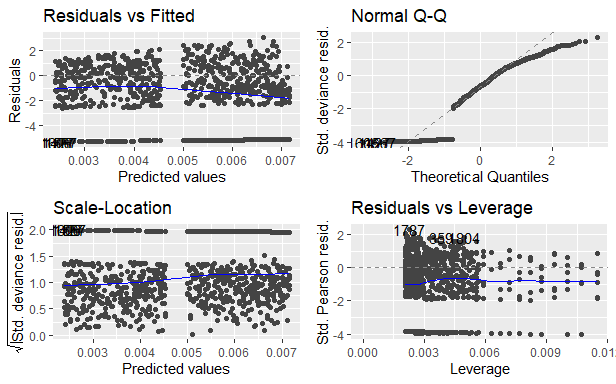


The effect sizes and confidence intervals for all the GLM outputs can be found in the table below:

|  | **Intercept** | **Day** | ***Lower CI*** | ***Upper CI*** | **Surgery** | ***Lower CI*** | ***Upper CI*** |
| --- | --- | --- | --- | --- | --- | --- | --- |
| **Distance Traveled** | 1.93 | 0.02 | *0.01* | *0.02* | -0.13 | *-0.34* | *0.08* |
| **Home Duration** | 0.05 | 0 | *0* | *0* | -0.01 | *-0.01* | *0* |
| **Significant Location Count** | 0.72 | 0.01 | *0* | *0.01* | -0.16 | *-0.26* | *-0.07* |
| **Significant Location Entropy** | 4.32 | -0.04 | *-0.06* | *-0.03* | 1.82 | *1.2* | *2.48* |
| **Steps** | 0 | 0 | *0* | *0* | 0 | *0* | *0* |
